# Prolonged preoperative fasting induces postoperative insulin resistance by ER-stress mediated Glut4 down-regulation in skeletal muscles

**DOI:** 10.7150/ijms.52701

**Published:** 2021-01-11

**Authors:** Ming-Wei Lin, Chih-I Chen, Tzu-Ting Cheng, Chien-Chi Huang, Jen-Wei Tsai, Guan-Ming Feng, Tzer-Zen Hwang, Chen-Fuh Lam

**Affiliations:** 1 Department of Medical Research, E-Da Hospital/E-Da Cancer Hospital, Kaohsiung, Taiwan.; 2 Department of Nursing, I-Shou University College of Medicine, Kaohsiung, Taiwan.; 3 Regenerative Medicine and Cell Therapy Research Center, Kaohsiung Medical University, Kaohsiung, Taiwan.; 4 Colorectal Surgery, Department of Surgery, E-Da Hospital/E-Da Cancer Hospital, Kaohsiung, Taiwan.; 5 Department of Anesthesiology, E-Da Hospital/E-Da Cancer Hospital, Kaohsiung, Taiwan.; 6 Department of Pathology, E-Da Hospital, Kaohsiung, Taiwan.; 7 Department of Plastic Surgery, E-Da Hospital, Kaohsiung, Taiwan.; 8 Department of Otolaryngology, E-Da Hospital, Kaohsiung, Taiwan.; 9 School of Medicine, I-Shou University College of Medicine, Kaohsiung, Taiwan.

**Keywords:** endoplasmic reticulum stress, fasting, insulin resistance, protein catabolism, skeletal muscle

## Abstract

Preoperative fasting aims to prevent pulmonary aspiration and improve bowel preparation, but it may induce profound systemic catabolic responses that lead to protein breakdown and insulin-resistant hyperglycemia after operation. However, the molecular mechanisms of catabolic reaction induced by prolonged preoperative fasting and surgical stress are undetermined. In this study, anesthetized rats were randomly assigned to receive a sham operation or laparotomy cecectomy. Fasting groups were restricted from food and water for 12 h before operation, while the feeding group had free access to food throughout the study period. Twenty-four hours after operation, the animals were sacrificed to collect blood samples and soleus muscles for analysis. Postoperative blood glucose level was significantly increased in the fasting group with elevated serum insulin and C-peptide. Continuous feeding reduced serum myoglobin and lactate dehydrogenase concentrations. Preoperative fasting activated inositol-requiring transmembrane kinase/endoribonuclease (IRE)-1α and c-Jun N-terminal kinase (JNK) mediated endoplasmic reticulum (ER)-stress, and reduced glucose transporter type 4 (Glut4) expression in the soleus muscle. Phospholamban phosphorylation was reduced and intracellular calcium levels were increased in the isolated skeletal muscle cells. Similar results were found in ER stress-induced C1C12 myoblasts. The expression of Glut4 was suppressed in the stressed C1C12, but was potentiated following inhibition of ER stress and chelation of intracellular free calcium. This study provides evidence demonstrating that prolonged preoperative fasting induces ER stress and generates insulin resistance in the skeletal muscle through suppression of Glut4 and inactivation of Ca^2+^-ATPase, leading to intracellular calcium homeostasis disruption and peripheral insulin resistance.

## Introduction

Perioperative fasting remains a common clinical practice in surgical patients. The aim of perioperative fasting is to prevent pulmonary aspiration during anesthesia induction, improve bowel preparation, and ameliorate the development of postoperative nausea/vomiting or other surgical-related complications 1, 2. However, prolonged fasting during the perioperative period has been shown to induce profound systemic catabolic responses. Clinical studies have found perioperative fasting aggravates systemic glycogen mobilization, protein breakdown, and resistance to the hypoglycemic effects of insulin 3-5. One study found skeletal muscle protein breakdown accounted for 20% of the weight loss at 2 weeks after major operations, and these patients required an additional 2 weeks to regain preoperative muscle function 6. Perioperative hyperglycemia has also been found to be associated with the development of postoperative surgical site infection, re-intervention, cardiac arrest, and death in diabetic and non-diabetic patients 7, 8. Therefore, current clinical practice guidelines recommend minimizing the duration of fasting in surgical patients pre- and post-surgery as well as provide preoperative carbohydrate loading in order to attenuate postoperative insulin resistance, nitrogen and protein losses, and enhance early recovery after operation 9, 10.

Protein breakdown and impaired glucose uptake in skeletal muscles have been shown to mediate muscle wasting and insulin resistance following surgical stress 11. However, the current understanding of perioperative intermediary metabolism in skeletal muscles considers only surgery-related factors 3, and does not take into account the combined catabolic challenges of fasting and surgical stress. Furthermore, very few preclinical animal models have examined the synergistic effects of dietary restrictions on the surgical-induced stress response 5.

Skeletal muscle ER tightly regulates whole body muscle proteostasis and calcium homeostasis through activation of the unfolded protein reaction (UPR) during severe skeletal muscle injury, myogenic differentiation, or regeneration 12, 13. Therefore, this study aimed to investigate skeletal muscle generation of ER stress and molecular regulation in response to laparotomy cecectomy with and without preoperative fasting in rats. We hypothesized that prolonged preoperative fasting superimposed ER stress during major surgical stress, thereby enhancing protein catabolism and reducing peripheral glucose uptake in distant skeletal muscles.

## Materials and Methods

### Animals and treatment protocols

The animal studies were conducted in compliance with the Animal Center of the E-Da Hospital and approved by the Institutional of Animal Care and Use Committee. Adult male adult Sprague-Dawley rats (250-300 g) were randomly assigned to one of three groups, the sham operation+fasting (Control), cecectomy+fasting (CFast) and cecectomy+feeding (CFeed) groups. The animals were housed in the laboratory animal center, which is a specific-pathogen-free environment with the standard 12:12 light-dark cycle. Animals assigned to fasting treatment were food and water restricted from 12 h before operation. The animals in the feeding group had free access to food and water throughout the perioperative period. All animals were housed in single in a transparent plastic cage after operation. The sham operation or cecectomy was performed under general anesthesia by intraperitoneal injection of cocktail anesthetic Zoletil^®^ (30 mg/kg). All anesthetized animals received laparotomies to expose the intestines. The sham operation group received only the laparotomy. In the cecectomy group, the cecum was identified and partially resected at the jejunum and ascending colon junction. The abdominal wall was then closed in layers and the animals were returned to their original cages after recovery from anesthesia.

Twenty-four hours post-operation, the animals were deeply anesthetized using inhaled isoflurane (3% v/v in oxygen) and blood samples were collected by direct cardiac puncture. Bilateral soleus muscles were harvested after euthanasia for analysis. A total of 65 rats were used in this study.

### Serum biochemical analysis

Serum concentrations of blood glucose, myoglobin, lactate dehydrogenase (LDH), and creatinine phosphokinase (CPK) were determined using an autoanalyzer (Quik-Lab®, Heraeus Electro-Nite International NV, Houthalen, Belgium). C-peptide and insulin levels in the serum samples were analyzed using commercially available ELISA kits (Mercodia AB, Uppsala, Sweden). Aldolase activity in the skeletal muscles was measured by a colorimetric assay kit (Abcam Biochemicals, Cambridge, MA).

### Measurement of intracellular calcium in isolated skeletal muscle cells

Soleus muscles were weighed and the outermost connective tissues were removed. A commercially available murine skeletal muscle dissociation kit was used to isolate the skeletal muscle cells (Miltenyi Biotec, Auburn, CA) 16. After resuspension in phosphate-buffered saline (PBS), the isolated skeletal muscle cells were incubated with Fluo-4 AM (5 μM, Thermo Fisher Scientific, Waltham, MA) for 30 min at room temperature. The intracellular calcium concentration level in isolated skeletal muscle cells was analyzed using a flow cytometer (FACS Calibur, BD Bioscience, San Jose, CA).

### Culture skeletal muscle myoblasts and in vitro ER stress experiments

Mouse skeletal muscle cell line (C1C12 myoblasts; ATCC^®^, Manassas, VA) were maintained in Dulbecco's modified Eagle's medium (DMEM) supplemented with 10% fetal bovine serum. When cells reached confluence, the medium was changed to the differentiation medium containing DMEM and 2% horse serum. After 10 additional days, the differentiated C2C12 cells fused into myotubes. ER stress was induced by culturing the differentiated C1C12 myoblasts in thapsigargin (TG) solution (1 μM; Sigma Aldrich, St. Louis, MO) for 48 h. Sodium phenylbutyrate (SP; an ER stress inhibitor, 1 μM; Sigma Aldrich, St. Louis, MO) and BAPTA-AM (an intracellular calcium chelator, 20 μM; Sigma Aldrich, St. Louis, MO) were pretreated 30 min before induction of ER stress in the C1C12 myoblasts. C1C12 cells were harvested at the end of experiments and analyzed for protein expressions.

### Histology and immunohistostaining

Muscle tissue were fixed in 10% buffered formal saline for at least 24 h. Biopsies were processed through increasing grades of alcohol and embedded in paraffin wax. Sections of skeletal muscle were stained using the Hematoxylin & Eosin staining method and examined under a light microscope. Skeletal muscle fiber diameters were measured under high-power fields (400X). Four randomly selected fields on each tissue section were observed by a pathologist, who was blinded to the treatment groups.

### Western blots

Muscle biopsies or cell lysate were minced and homogenized in a lysis buffer. Equal amounts of total protein (50-100 μg) were loaded into polyacrylamide gels (9-12%). The protein was then transferred onto nitrocellulose membranes through the wet-transfer method. The membranes were incubated overnight with primary antibodies of appropriate dilutions (1:1000) at 4ºC. After washing with a phosphate buffer solution, the membranes were incubated with appropriately diluted horseradish peroxidase-linked secondary antibodies for 1 h at room temperature. Densitometries of the Western blots and densities of immunofluorescence were quantified by the software provided by the Image J (1.48v, National Institutes of Health, USA). The primary antibodies were purchased from the Cell Signaling (Denver, MA; for Glut4, JNK, phospholamban and Caspase-3), the GeneTex (Irvine, CA; for binding immunoglobulin protein) and the Abcam Biochemicals, (Cambridge, MA; for IRE-1α).

### Statistical analysis

Results are presented as the mean±SD. Data were compared by ANOVA followed by multiple group comparisons using Tukey test. Statistical significance was accepted at a level of *P*< 0.05. All of the statistical analyses were performed using the Sigmaplot software (version 14; Systat Software Inc., San Jose, CA, USA).

## Results

### General outcomes and serum biochemical analysis

**Figure 1:**
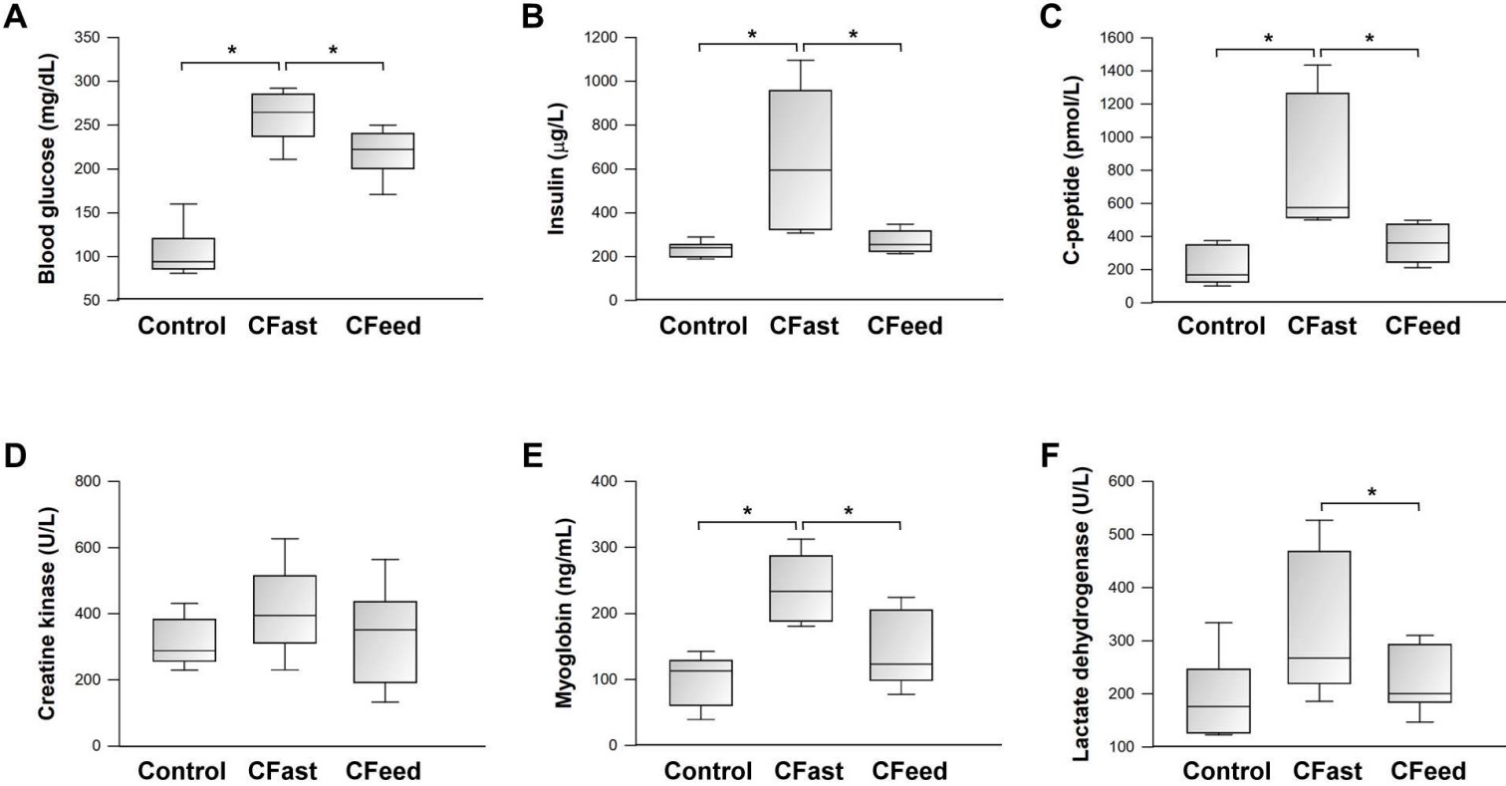
Serum biochemical analysis. Results were analyzed using the one-way ANOVA. Data are presented as mean ± SD. *P<0.05; n=5-7 different animals in each group.

There was no perioperative mortality in any of the three experimental groups. Body weight loss (body weight difference pre- and 24-h post-operation) was not different between the CFast and CFeed groups (8.9±3.6 g vs 6.7±3.2 g, respectively; P=0.253, n=7-9). In comparison to the sham-operation group, preoperative fasting significantly increased blood glucose levels at 24 h after laparotomy cecectomy (CFast) (Figure 1A). The postoperative hyperglycemic response was associated with significantly elevated serum levels of insulin and C-peptide (Figure 1B-C), indicating the development of insulin-resistant hyperglycemia. Although postoperative blood glucose levels were also significantly increased in animals assigned to continuous oral feeding before operation (CFeed) (Figure 1A), the serum concentrations of insulin and C-peptide were suppressed to levels similar to that of the sham-operation group (Figure 1B-C). Serum biochemical markers (CPK, myoglobin and LDH) were analyzed for evidence of skeletal muscle breakdown. Myoglobin and LDH levels were significantly increased in the CFast group, but not in the CFeed animals (Figure 1E-F).

### Histological examination and glycolytic activity in skeletal muscle

**Figure 2:**
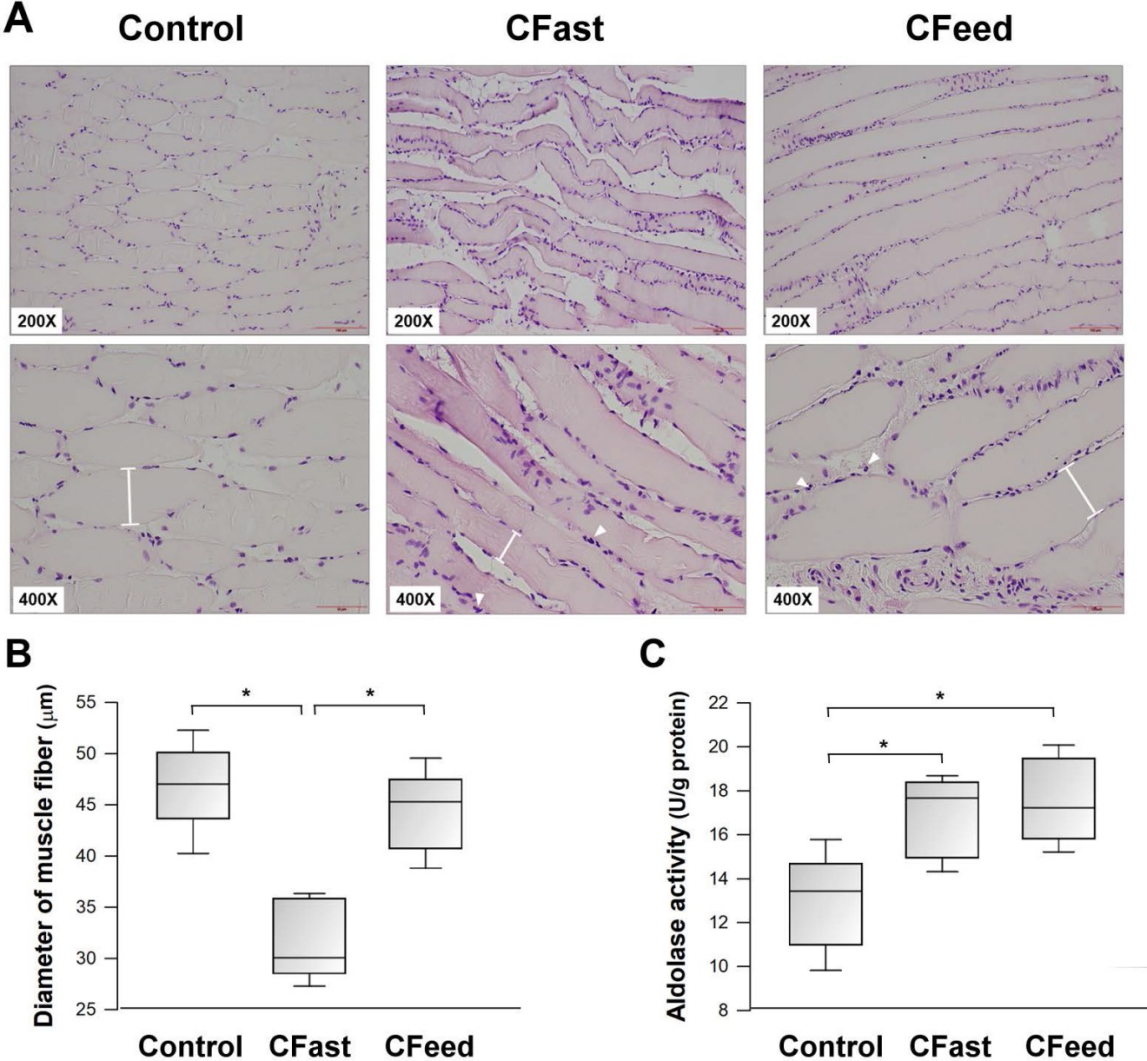
(A) Representative Hematoxylin & Eosin staining histologic sections of soleus muscles (200x and 400x magnifications). Higher cellularity was found in the tissue surrounding muscle fibers. Arrowheads indicate the infiltration of polymorphoneuclear cells. The diameters of muscle fibers were measured (I-shapes). (B) The diameters of muscle fibers were compared between the three groups. *P<0.05; n=7 different tissue sections in each group. (C) The enzymatic activity of aldolase in the soleus muscles. *P<0.05; n=5-7 different animals in each group. Results were analyzed using the one-way ANOVA and data are presented as mean ± SD.

The mean diameter of the soleus muscle fibers was significantly smaller in the CFast group (31.3±3.3 μm vs 46.7±3.8 μm vs 44.4±3.5 μm for the CFast, SFast and CFeed groups, respectively; P<0.001). Compared with the sham-operation group, there was increased infiltration of mononuclear cells in the skeletal muscle in the animals who received cecectomies (CFast and CFeed groups) (Figure 2A-B). Tissue glycolytic enzyme activity (aldolase activity) was also significantly increased in the soleus muscle harvested from animals received cecectomy (CFast and CFeed groups) (Figure 2C).

### Expressions of glucose transporter and unfolded protein response (UPR) in skeletal muscle

**Figure 3:**
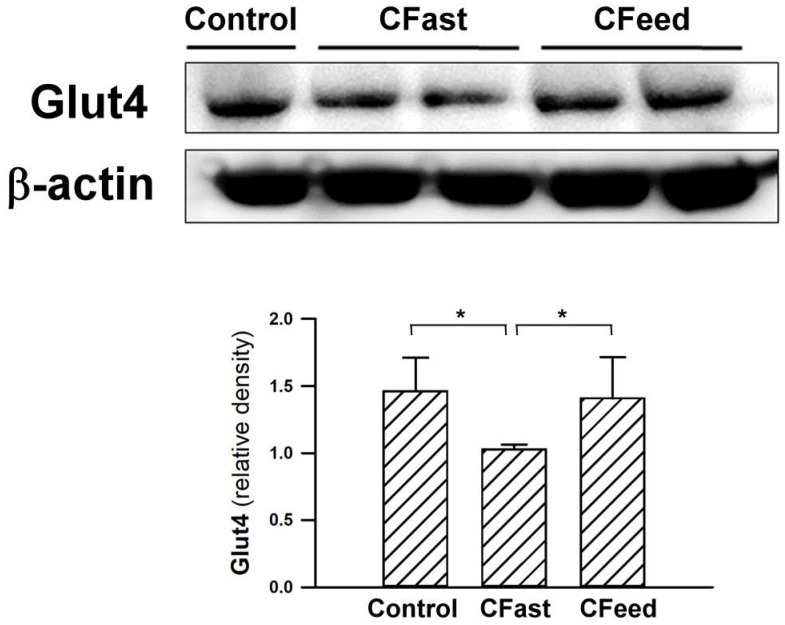
Protein expressions of Glut4 in the soleus muscles. Results were analyzed using the one-way ANOVA and data are presented as mean ± SD. *P<0.05; n=4-5 different animals in each group.

**Figure 4:**
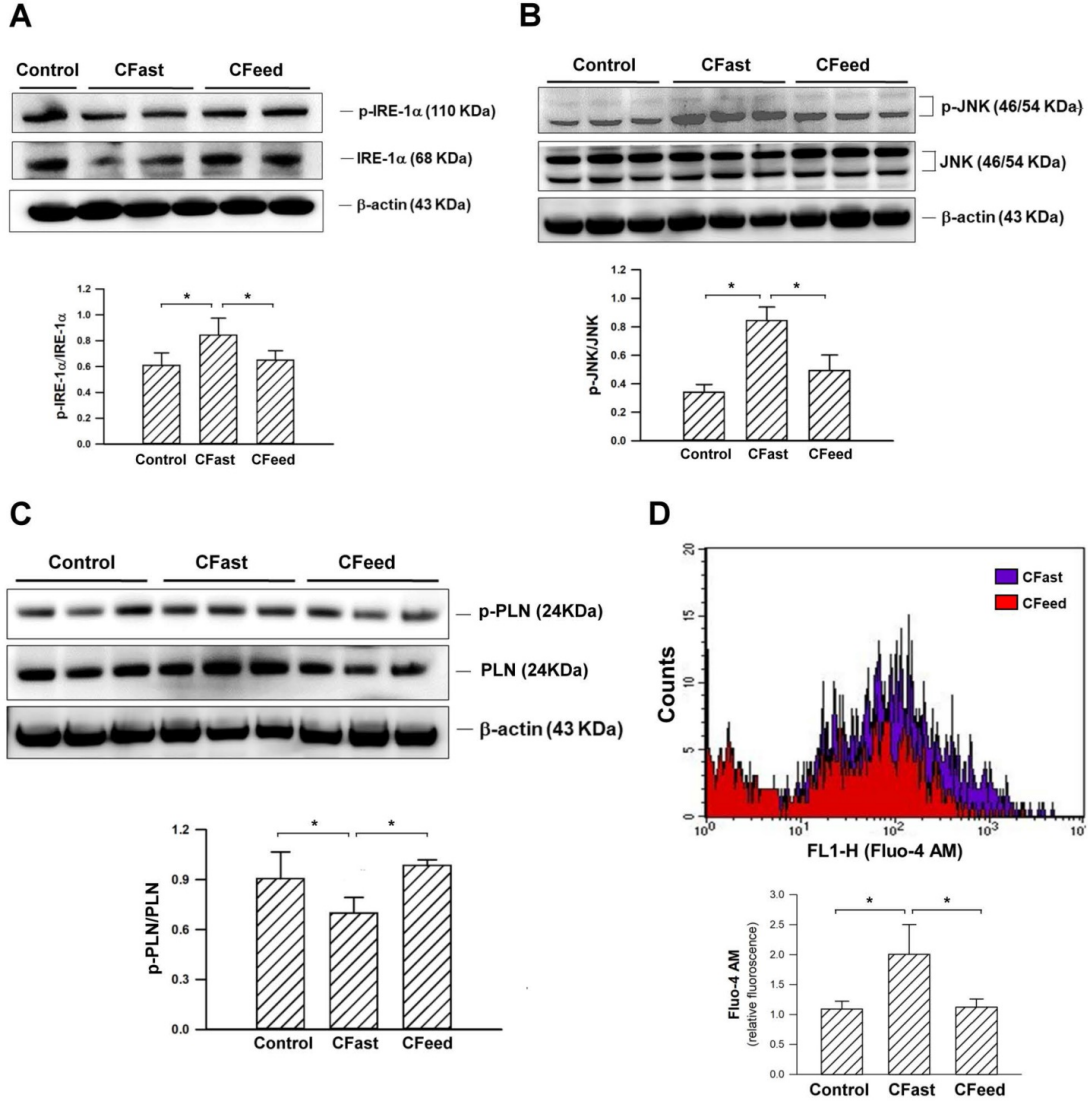
(A) Protein expressions of IRE1α and p- IRE1α in the soleus muscles. Results were analyzed using the one-way ANOVA and data are presented as mean ± SD. *P<0.05; n=4-5 different animals in each group. (B) Protein expressions of JNK and p-JNK in the soleus muscles. Results were analyzed using the one-way ANOVA and data are presented as mean ± SD. *P<0.05; n=4-5 different animals in each group. (C) Expressions of the pump regulator PLN in the soleus muscles. Results were analyzed using the one-way ANOVA and data are presented as mean ± SD. *P<0.05; n=4-5 different animals in each group. (D) Measurement of intracellular calcium in the freshly isolated skeletal muscle cells of soleus muscle using flow cytometry. Significantly higher Flou-4 AM fluorescence density was detected in the muscle cells of CFast group. Results were analyzed using the one-way ANOVA and data are presented as mean ± SD. *P<0.05; n=5 different animals in each group.

Tissue expression of Glut4 (an important insulin-regulated glucose transporter in skeletal muscles) 14, 15 were significantly suppressed in rats assigned to preoperative fasting for 12h before cecectomy. The protein levels of Glut4 in the soleus muscle were restored in the CFeed group (Figure 3). In order to determine whether skeletal muscle was under ER stress in CFast groups, the effectors of UPR, IRE-1α, was analyzed. The phosphorylated form of IRE-1α (p-IRE-1α/total IRE-1α) in the soleus muscle was enhanced in the CFast group (Figure 4A). An analysis of the downstream IRE-1α signaling cascade found the JNK protein was significantly phosphorylated in the rats subjected to preoperative fasting (Figure 4B). The phospholamban (PLN) phosphorylation was significantly suppressed in the soleus muscle of CFast rats (Figure 4C). These results suggested that the skeletal muscle in CFast groups was under ER stress.

### Measurement of intracellular calcium in isolated skeletal muscle cells

Intracellular calcium in the freshly isolated skeletal muscle cells of soleus muscle were measured using flow cytometry. The analysis showed that Fluo4-AM labeled intracellular calcium concentration was significantly enhanced in the CFast animals (Figure 4D). However, the intracellular calcium level was unchanged in CFeed group compared to sham control group. The results suggested that the calcium homeostasis in CFast group was disrupted.

### Regulation of Glut4 expression in cultured myoblasts under ER stress

**Figure 5:**
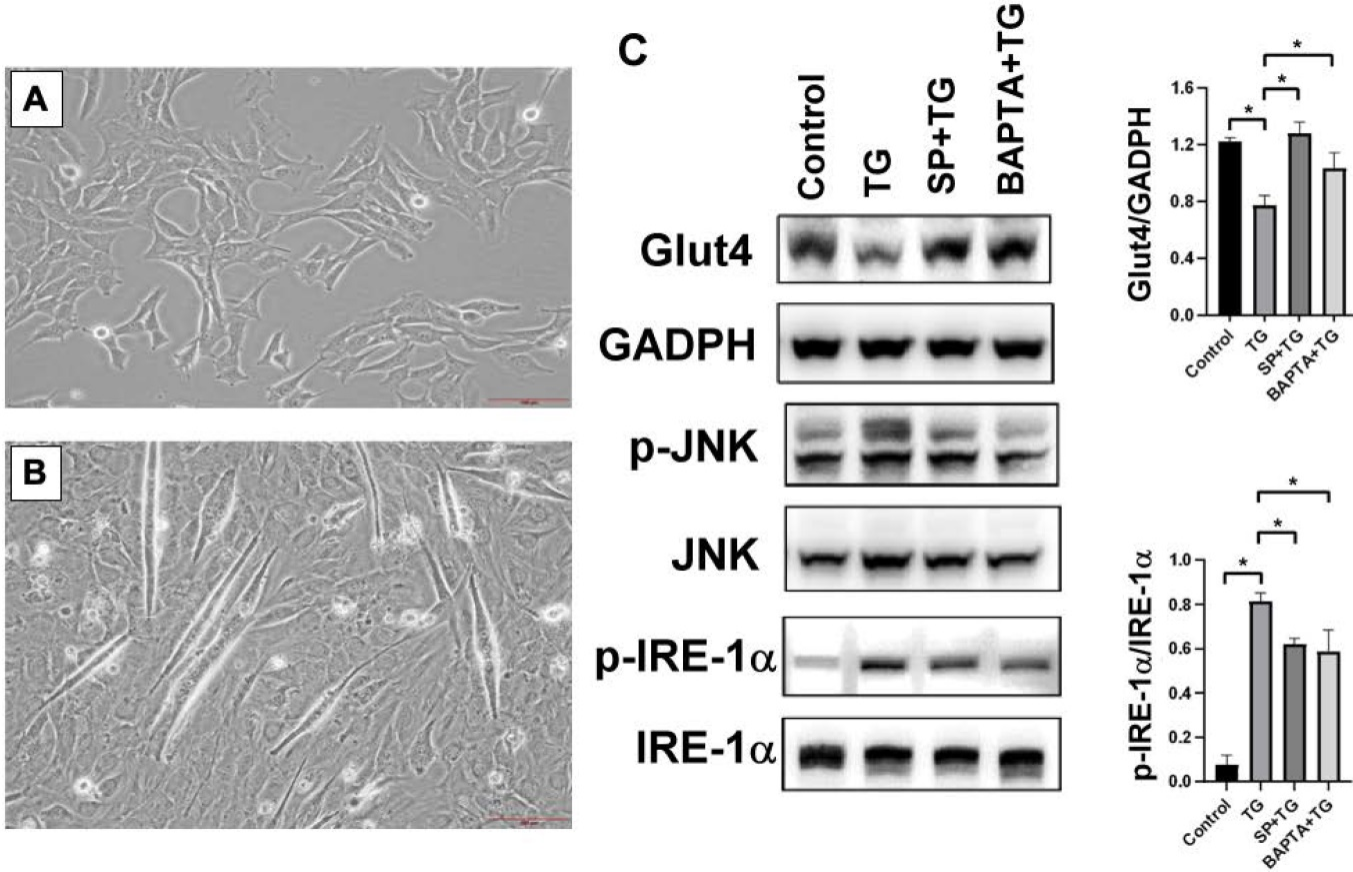
(A) The undifferentiated mouse C1C12 skeletal muscle myoblasts were cultured in the DMEM, (B) and the myoblasts became differentiated and fused to form multinuclear myotubes following incubation in DMEM containing 10% fetal bovine serum for 10 days. (C) ER stress was induced in the differentiated C1C12 myoblasts by treatment with a SERCA pump inhibitor (TG, 1μM), and resulted in significant suppression of Glut4. After pretreatment with an ER stress inhibitor sodium phenylbutyrate (SP, 1μM) or intracellular calcium chelator (BAPTA-AM, 20μM), the expressions of Glut4 and the ratio of p-IRE-1α/IRE-1α were significantly restored. Results were analyzed using the one-way ANOVA and data are presented as mean ± SD. *P<0.05; n=5 different experiments.

In order to determine the mechanisms of ER stress-mediated Glu4 downregulation, excessive ER stress was induced in mouse C1C12 myoblasts by a SERCA inhibitor, TG 17. After culturing in differentiation medium for 10 days, the proliferative C1C12 (Figure 5A) differentiated and fused to form multinuclear myotubes (Figure 5B). The protein expression of Glut4 was significantly suppressed and phosphorylated IRE-1α was enhanced in the differentiated C1C12 cells after ER stress induction by TG. Following pretreatment with ER stress inhibitor, SP, or intracellular calcium chelator, BAPTA-AM, the expression of Glut4 was upregulated and the phosphorylation level of JNK was reduced in the stressed C1C12 myoblasts (Figures 5C), suggesting that Glut4 downregulation in the skeletal muscle cells was associated with JNK signaling-mediated ER stress induction and intracellular calcium homeostasis disruption.

## Discussion

Clinical observational studies have demonstrated that prolonged fasting during the perioperative period leads to the development of insulin-resistant hyperglycemia and systemic skeletal muscle wasting after major operations 18. However, the underlying mechanism of this surgery-related catabolic reaction remains undetermined. Our study characterized an experimental model of systemic catabolism due to the synergistic effects of preoperative fasting and surgical stress in rats. We found that these animals developed insulin-resistant hyperglycemia and skeletal muscle breakdown after operation. The analysis of the molecular changes in the peripheral calf muscles showed that the combination of preoperative fasting and cecectomy enhanced the IRE-1α/JNK-dependent UPR in the muscle tissue, and impaired Glut4-related glucose transportation into muscle cells. In addition, the increased ER stress attenuated the phosphorylation of PLN in the SERCA, which in turn, increased the cytosolic calcium concentrations in the skeletal muscle cells. Intracellular calcium overload-mediated ER stress and suppression of Glut4 expression eventually precipitated the development of peripheral insulin-resistant hyperglycemia following prolonged preoperative fasting and major surgical stress.

This study characterized a simple experimental model of a cecum resection that carried a very low perioperative mortality rate, but it induced clinically comparable postoperative catabolic reactions in the animals with prolonged preoperative fasting. In the fasting group, the blood glucose level was significantly elevated at 24 h post cecectomy. The postoperative hyperglycemia was associated with significantly higher serum concentrations of insulin and C-peptide, which is consistent with the development of postoperative insulin-resistance in patients receiving major abdominal surgery 18, 19. We also analyzed the serum markers of muscle enzymes, histological changes and aldolase activity in the soleus muscle to characterize skeletal muscle injury or muscle wasting after cecectomy, as postoperative muscle loss significantly impacts morbidity and mortality in patients undertaking major open abdominal surgery 20. In addition to enhanced infiltration of inflammatory cells, the increased activity of aldolase, an important adenosine 5'-monophosphate-activated protein kinase (AMPK)-regulated stress sensor of glucose availability 21, in the soleus muscle of rats received cecectomy (both CFast and CFeed groups) indicated the development of systemic stress response following major operation. However, our results showed that the serum levels of myoglobin and LDH were more significantly elevated, and the muscle fiber diameter of the soleus muscles were also significantly smaller in the rats assigned to fasting before cecectomy. These serum biochemical and morphological changes clearly indicated that systemic protein catabolic reactions and skeletal muscle wasting was due to preoperative fasting. Although the animals that had free access to chow and water before cecectomy also developed hyperglycaemia, the serum levels of insulin and C-peptide were significantly reduced. Furthermore, plasma muscle markers and muscle fiber diameter were also restored in the feeding group. Improved insulin resistance and skeletal muscle catabolism after open abdominal surgery in this murine experimental model supported the clinical evidence that the avoidance of prolonged preoperative fasting and oral carbohydrate loading may reduce insulin resistance and skeletal muscle protein catabolism. This in turn, can create a positive impact on perioperative glucose control and muscle preservation after major operations 22. The validation of this experimental model suggests that further investigation into the molecular mechanisms of postoperative insulin resistance and skeletal muscle catabolism is warranted.

Prolonged fasting and extensive bowel resection generates substantial systemic ER stress, extending to the remote skeletal muscles. In order to alleviate the stress, the ER activated a signaling network, namely the UPR, to restore homeostasis in the skeletal muscles 12. Under basal conditions, IRE-1α are bound to the ER chaperone GRP78 to maintain an inactive state 12. GRP78 is a major ER chaperone protein that regulates the UPR as well as mediate anti-apoptotic properties 23. The combination of preoperative fasting and cecectomy generated systemic ER stress that separated the bonds between UPR enzymes and GRP78, eventually activating the downstream pathways of IRE-1α and leading to transient suppressed protein translation, increased ER protein-folding chaperones, and enhanced ER-associated protein degradation 24. Phosphorylation of IRE-1α activates proper folding and increases ER-associated protein degradation 25. However, prolonged or substantial hyperglycemic conditions may increase IRE-1α kinase activity and activate the down-stream JNK pathway, leading to insulin resistance 26, 27 and skeletal muscle remodeling 15. Our results showed that there was significantly higher ER stress and UPR in the soleus muscle harvested from the CFast group, as demonstrated by increased phosphorylation of the IRE-1α/JNK signaling pathway.

**Figure 6:**
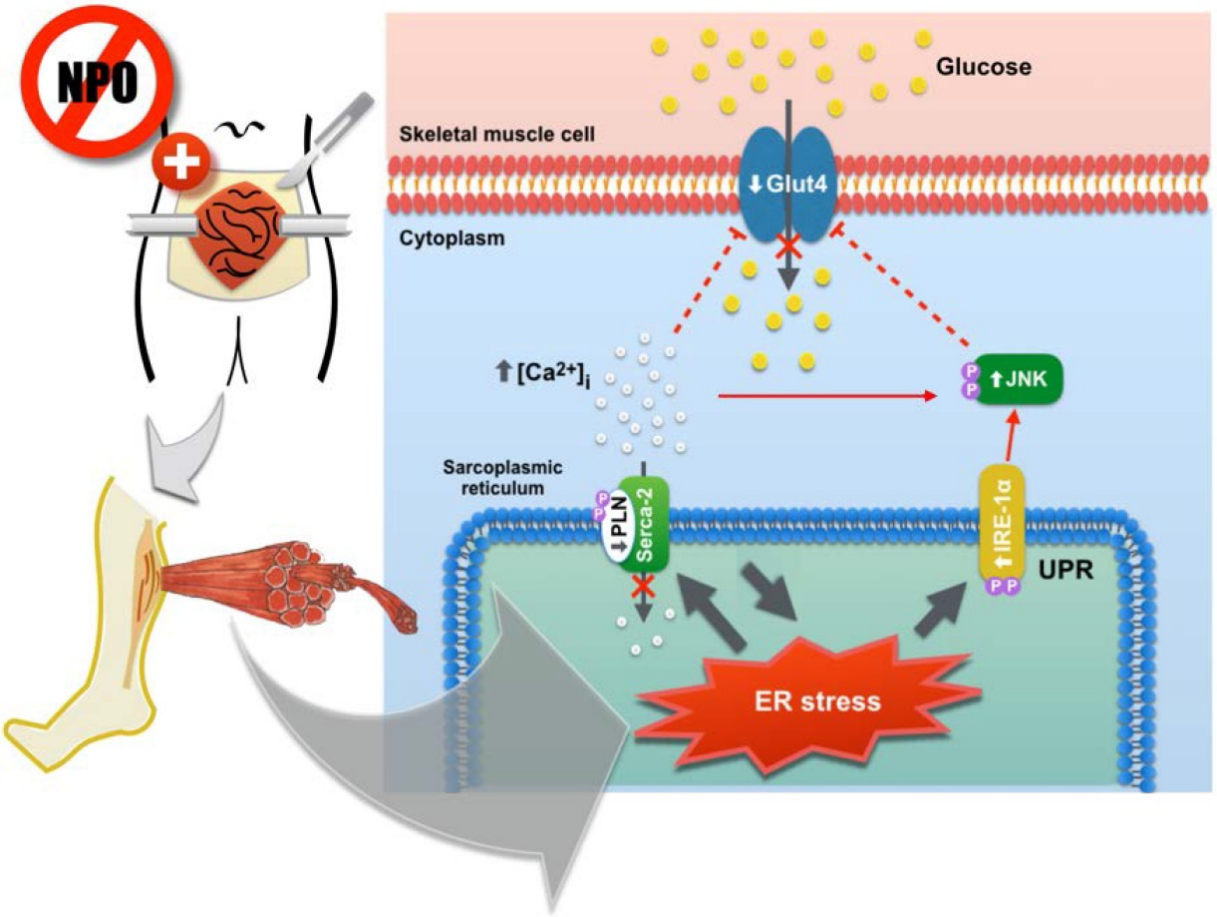
Diagram demonstrating the molecular mechanisms of prolonged preoperative fasting and surgical stress in development of postoperative insulin-resistant hyperglycemia and unfolded protein response in the peripheral skeletal muscles. This “2-hit” model induces ER stress in the skeletal muscles and in turn, suppresses muscular glucose transportation by attenuating Glut4 expression and increasing intracellular Ca^2+^ loading. Elevated ER stress also promotes skeletal muscle protein breakdown through the activation of IRE-1α/JNK pathway.

Skeletal muscles play a fundamental role in the whole body glucose homeostasis by transportation of blood glucose into skeletal muscle cells through the insulin-sensitive glucose transporters 28. Glut4 is the main subtype of glucose transporter for muscular glucose. In skeletal muscle cells, Glut4 is stored within the intracellular pools as an inactive form 14. During hyperglycemic or stress conditions, insulin initiates a signaling phosphorylation cascade which rapidly translocate Glut4 from inactive storage sites to the plasma membrane and enhances muscular uptake of glucose, leading to reduced blood glucose levels 29. Ca^2+^ influx is also an important factor in regards to insulin-mediated glucose uptake and Glut4 translocation onto the plasma membrane in skeletal muscles 30. The SERCA pump is a major regulator of muscular glucose transport in skeletal muscle 31. The activity of SERCA is modulated by an integral membrane protein of the sarcoplasmic reticulum, phospholamban, that transports Ca^2+^ from cytosol into sarcoplasmic reticulum to induce muscle relaxation and transportation of muscle glucose 32. Under certain pathological conditions, the generation of ER stress can suppress SERCA expression and activity, causing impaired glucose metabolism and insulin resistance development 33. ER stress is an important mediator of insulin resistance in skeletal muscle 34. Glut4 downregulation and insulin resistance were observed after calcium homeostasis disruption and ER stress induction 35,36. In our study, we theorized that the perioperative “2-hit” stress (fasting and cecectomy) impaired SERCA activity in the skeletal muscle by attenuating PLN phosphorylation, thereby increasing cytosolic Ca^2+^ concentrations and reducing muscular glucose transportation. In the in vitro experiments, our analysis also found that the expression of Glut4 was reduced in cultured C1C12 myoblasts that were stressed by SERCA pump inhibition. By feeding the animals before open cecectomy, we found that the ER stress in the soleus muscle was suppressed, as IRE-1α/JNK signaling of UPR were restored to similar levels compared to the sham operation group. The coupling of phosphorylated phospholamban and SERCA-2 complex stabilized intracellular Ca^2+^ homeostasis. In conjunction with Ca^2+^ homeostasis, the upregulation of insulin-sensitive Glut4 facilitated the transportation of glucose into the skeletal muscle cells and improved tissue sensitivity to insulin stimulation (Figure 6). These mechanistic improvements not only attenuated the development of postoperative insulin-resistant hyperglycemia, the suppression of UPR in the remote skeletal muscle also reduced protein catabolism and muscle wasting after surgery.

In conclusion, we demonstrated that prolonged preoperative fasting induces ER stress in peripheral skeletal muscles and generates insulin resistance in the skeletal muscle through muscular glucose transportation suppression by using a rodent model of “2-hit” perioperative stress. Elevated ER stress promotes skeletal muscle UPR, leading to protein breakdown. Continuous oral feeding throughout the preoperative period significantly improved postoperative peripheral insulin-resistance and attenuated protein catabolic reaction in the skeletal muscle, both which are clinically important in enhancing the patient recovery after major surgery.
